# Differences in Molecular Adsorption Emanating from the (2 × 1) Reconstruction of Calcite(104)

**DOI:** 10.1021/acs.jpclett.2c03243

**Published:** 2023-02-16

**Authors:** Jonas Heggemann, Yashasvi S. Ranawat, Ondřej Krejčí, Adam S. Foster, Philipp Rahe

**Affiliations:** † Fachbereich Physik, Universität Osnabrück, 49076 Osnabrück, Germany; ‡ Department of Applied Physics, Aalto University, Helsinki FI-00076, Finland; § Nano Life Science Institute (WPI-NanoLSI), Kanazawa University, Kanazawa 920-1192, Japan

## Abstract

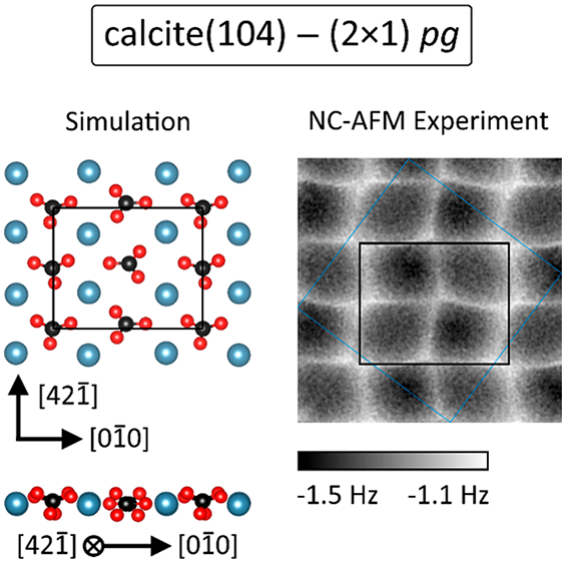

Calcite, in the natural environment the most stable polymorph of calcium carbonate (CaCO_3_), not only is an abundant mineral in the Earth’s crust but also forms a central constituent in the biominerals of living organisms. Intensive studies of calcite(104), the surface supporting virtually all processes, have been performed, and the interaction with a plethora of adsorbed species has been studied. Surprisingly, there is still serious ambiguity regarding the properties of the calcite(104) surface: effects such as a row-pairing or a (2 × 1) reconstruction have been reported, yet so far without physicochemical explanation. Here, we unravel the microscopic geometry of calcite(104) using high-resolution atomic force microscopy (AFM) data acquired at 5 K combined with density functional theory (DFT) and AFM image calculations. A (2 × 1) reconstruction of a *pg*-symmetric surface is found to be the thermodynamically most stable form. Most importantly, a decisive impact of the (2 × 1) reconstruction on adsorbed species is revealed for carbon monoxide.

Besides its occurrence in the natural environment as the principal constituent of limestone and marble,^1^ calcite is broadly used as a construction material,^2^ is employed in agricultural soil and water treatment,^3^ and is currently investigated as both an adsorbent for pollutants^4^ and a capture material for CO_2_.^5^ Furthermore, calcite commonly forms the inorganic phase of biominerals^6^ such as the shells of mollusks or teeth of sea urchins.^7^

The (104) face is the most stable cleavage plane of calcite^8,9^ and supports virtually all processes that involve the material calcite.^10^ The bulk-truncated surface expresses a rectangular (1 × 1) unit cell with dimensions of 0.5 nm × 0.81 nm, contains two calcium atoms and two carbonate groups per unit cell,^8^ and belongs to the two-dimensional crystallographic group (planar space group) *pg* with a glide plane reflection *g* as the symmetry element. The symmetry properties are established to a large degree by the different orientations of the carbonate groups. In particular, the *g* symmetry operation is defined by a reflection (*m*) and a translation (*t*) along the axes of glide reflection parallel to the  direction; the translation distance amounts to half the unit cell size.^11^ For the (1 × 1) surface, the axes of glide reflection are located on either the calcium atom or carbonate group rows.

Early experimental studies of the calcite(104) surface have suggested the presence of two modifications that are not in agreement with the bulk-truncated (1 × 1) surface structure: the so-called row-pairing^12−17^ and (2 × 1) reconstruction.^13,15,18^ The appearance of row-pairing in atomic force microscopy (AFM) images gives hints of violation of the glide-plane reflection symmetry element which was, in contrast, not identified by X-ray reflectivity and diffraction experiments.^19−21^ Note that loss of the *g* symmetry element would render calcite(104) a chiral surface. Exciting as this would be in the context of the homochirality of life,^22^ this conclusion is in disagreement with the observed reactivity: the selective adsorption of l- and d-amino acids has been explained by the presence of chiral step edges.^23^ In turn, evidence for the (2 × 1) reconstruction was previously given from LEED^18,24^ and AFM^15^ experiments. In particular, it has been highlighted that the appearance in NC-AFM data can depend on the tip–sample interaction strength,^13^ likely explaining literature data where the (2 × 1) reconstruction has not been observed.^17^ Suggestions for an origin of the (2 × 1) reconstruction include an influence of step edges^8,25^ or of cation ordering.^26,27^ Still, the underlying formation mechanisms and the microscopic surface geometry remained a topic of investigation to date.

Here, we take advantage of the breakthroughs that functionalized tips have demonstrated in molecular imaging with non-contact atomic force microscopy (NC-AFM)^28^ and integrate it with our own preparation technique for imaging bulk insulating systems^29^ to resolve the surface structure of calcite(104). Benefiting from the resolution power of CO-functionalized NC-AFM tips, a (2 × 1) reconstructed surface is consistently found when imaging the surface with symmetric tips in ultrahigh vacuum and at 5 K. Furthermore, no violation of the *pg* symmetry element is evident. A comparison with density functional theory (DFT) calculations, including state-of-the-art dispersion corrections as well as AFM image simulations, confirms an alternating rotation of every second surface carbonate group along the  surface direction as the characteristic of the surface reconstruction. We find that this reconstruction is the thermodynamically most stable surface structure and, most importantly, generates two different adsorption sites for carbon monoxide molecules. The finding of different adsorption geometries within the (2 × 1) reconstructed surface unit cell for CO molecules at 5 K has important consequences when studying processes on calcite(104).

This Letter is organized as follows: First, we analyze experimental high-resolution NC-AFM image data and determine the symmetry properties. Second, we present the DFT results and use AFM image calculations to validate the structural model. Third, we investigate the adsorption characteristics of CO and find an influence of the (2 × 1) reconstruction.

Figure 1a presents an NC-AFM frequency-shift Δ*f* image of calcite(104), acquired in constant-height mode with a CO-terminated tip at 5 K. Prior to NC-AFM experiments, the calcite(104) surface was prepared by *in situ* cleaving (see the Supporting Information for further details). A periodic lattice with bright rows running along the main  and  surface directions is apparent in these image data, generated from sharp features that resemble high-resolution imaging of molecular structures with CO-terminated tips.^30^ Most importantly, a (2 × 1) reconstruction is clearly visible from the modulation along  of the width of every second bright row (see white markers in lower left corner). The second central features defining the calcite(104) surface lattice are the dark pores within the lattice structure. After the deposition of CO molecules, we can identify several protruding features that substitute these dark pores of the lattice; two examples are marked by dark green arrows in Figure 1a. Previously, CO has been found to adsorb exclusively on calcium,^31^ in agreement with our own DFT analysis and the Blyholder model for CO adsorption on metal surfaces.^32^ Thus, the dark pores are identified as the calcium sites and the alternating orientation of the bright linkers apparent at the lattice vertices reflect the position of the carbonate groups. Throughout this Letter, we denote the rows along  of the calcium atoms as A, A′ and the rows along  of carbonate groups as B, B′ (see also markers in Figure 1a,f). With this naming, we reflect the chemical difference of the two rows (capital letters) as well as the difference within the (2 × 1) unit cell (unprimed and primed). To ensure a symmetric and unmodified CO geometry, the CO tip was characterized in scanning tunnelling microscopy (STM) mode on CO/Ag(111) before (Figure 1d) and after (Figure 1e) NC-AFM measurements on calcite(104). While the image in Figure 1d is in excellent agreement with the previous analysis of STM-contrast of symmetric CO tips,^33^ the similarity between panels d and e highlights that the CO tip remained unchanged during the acquisition of Figure 1a on calcite(104).

**Figure 1:**
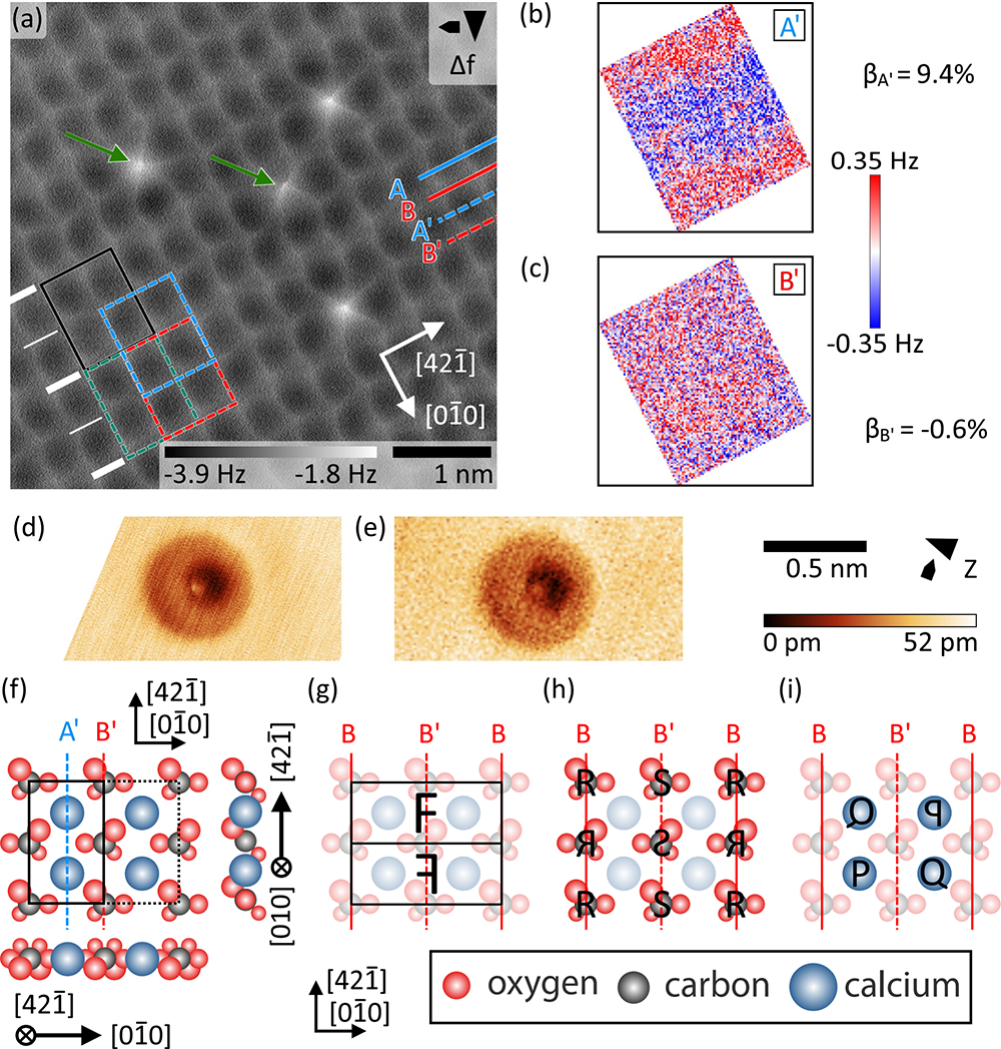
Symmetry properties of the (2 × 1) reconstructed calcite(104) surface. (a) Constant-height frequency-shift (Δ*f*) image acquired with a CO-terminated tip at 5 K. A (2 × 1) unit cell (black), two glide-reflected unit cells (red and blue), and a translated unit cell (green) are included in the lower left. Two CO molecules are marked by green arrows. (b and c) Results of the algorithmic symmetry test using the data marked in panel a by the (b) black and blue and (c) black and red rectangle. The plots show the deviation at each pixel within the (2 × 1) unit cell for the two possibilities of positioning the axes of glide reflection. The cases for axes located on rows A and B yield similar results (data not shown). (d and e) Reverse imaging of the CO tip using CO/Ag(111). Images were acquired (d) before and (e) after the calcite imaging experiments and are in good agreement with the shape of imaging a surface-adsorbed CO molecule with a CO tip as reported before.^33^ (f) Geometry of the bulk-truncated surface with the axes of glide reflection A and B as well as the (1 × 1) and (2 × 1) unit cell markers included. (g–i) Conclusions on the surface geometry of a (2 × 1) unit cell following from the presence of the *g* symmetry element. The upper and lower subcell of the (2 × 1) unit cell are related to each other by reflection as abstracted by the letter F. Letters P, Q, R, and S reflect the *g* symmetry relations for the (2 × 1) reconstructed surface. Experimental parameters: (a) *A* = 0.39 nm, (d and e) *U*_tip_ = 5 mV, *I*_t_ = 5pA. Fast and slow scan directions are indicated by left/right and up/down-pointing arrows, respectively, next to the image channel.

We identify a (2 × 1) reconstruction with a *g* symmetry element for the NC-AFM image in Figure 1a. This identification is based on the application of a specifically developed algorithmic test that quantifies the presence of a *g* symmetry element by comparing original and symmetry-processed data within single unit cells (see the Supporting Information for details). Here, this procedure is applied to the unit cell marked by the black rectangle in Figure 1a. The associated blue and red rectangles represent the reflected–translated unit cells with the symmetry axis located on either row A′ and B′, respectively. In turn, the green rectangle marks a translated unit cell, which is considered for determining the detection limit set by the experimental noise. As the final result, the algorithmic symmetry test delivers the minimum relative error  for an axis *X*, whereby the minimized RMS deviation Δ_min_^*pg*,*X*^ between an original and *g*-processed (2 × 1) unit cell as well as the minimized RMS deviation Δ_min_^*t*^ between data of an original and translated (2 × 1) unit cell are determined by allowing an optimization of the symmetry axis location (see the Supporting Information and Figure S2). For the data in Figure 1a, the *g* symmetry element is violated when the axes of glide reflection are positioned on rows A′ (calcium atom row, β_A′_ = 9.4%), while a match of the imaged surface structure is found when using axes B′ (carbonate group row, β_B′_ = −0.6%). The local deviation Δ_min_^*pg*,*X*^(*i*, *j*) within a (2 × 1) unit cell visualizes the difference between the original and *g*-processed unit cell (see Figure 1b,c). We consistently find the presence of the glide plane reflection symmetry element with β_B,B′_ =  in these NC-AFM data acquired with symmetric CO-terminated tips (see Figure S3 for further examples), while relative errors for the axes on rows A and A′ are larger by at least one order of magnitude. We have confirmed this result using a total of five representative NC-AFM images acquired with well-defined tips and with usually more than ten unit cells analyzed within each image. Relative errors are furthermore found to be substantially larger than zero, irrespective of the positioning of the symmetry axes, when asymmetric or blunt tips are used for imaging. Under these conditions, the apparent row-pairing reconstruction can be observed (see Figure S4). Thus, we conclude that the row-pairing effect is a tip artifact and the real calcite(104) surface belongs to the planar space group *pg* and is indeed (2 × 1) reconstructed.

The finding that the axes of glide reflection are consistently located on the rows of the carbonate groups allows us to derive conclusions on the surface structure solely from symmetry arguments. The sketch in Figure 1g shows a (2 × 1) unit cell separated into two subcells of the same size, with the axes of glide reflection located at the center and the edges of the (2 × 1) unit cell, running along the rows of the carbonate groups in the  direction. To satisfy the *g* symmetry element, first, the orientation of two carbonate group pairs adjacent in the  direction are each connected via the *g* symmetry element (abstracted by the characters R, 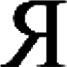, S, and 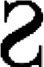 in Figure 1h), while the carbonate groups R and S are independent due to the (2 × 1) reconstruction. Effectively, the (2 × 1) reconstruction therefore induces rows along  at the carbonate group positions, which are alternating along the  direction. This conclusion is in full agreement with the experimental observation of a modulated row width (see also Figure 1a). Second, diagonally adjacent calcium atoms are related to each other by reflection as indicated by the letters P and 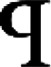, as well as Q and 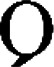 in Figure 1i; this diagonal relation is a consequence of the off-axis position of the calcium atoms. In contrast, the properties of the P and Q calcium atoms are again independent due to the (2 × 1) reconstruction. This relation effectively delivers a checkerboard-like pattern, which is also in agreement with the experimental findings in Figure 1a (see also Figure S3 for further examples).

To search for the atomic model of the reconstructed surface, we performed a systematic series of DFT calculations rotating and displacing carbonate groups in alternate rows of the (1 × 1) surface. Using this procedure, we found only one stable reconstruction with the geometric structure of the top layer shown in Figure 2a and 0.21 eV lower in energy than the (1 × 1) surface. From comparisons of DFT functionals with and without van der Waals interactions (see Table 1 for details), it is clear that van der Waals plays a decisive role in forming the (2 × 1) structure, which may explain why earlier DFT studies did not observe it. In contrast, when using PBE without dispersion correction, the (1 × 1) surface termination is slightly favored, with both the (1 × 1) and (2 × 1) terminations remaining stable. As shown in Figure 2a, the reconstruction is predominantly defined by a rotation of every second carbonate group along , and as a result, the two oxygen atoms of one carbonate group are brought to nearly the same vertical height. We note that the predicted structure is quite similar to that suggested by classical surface phonon calculations^34^ with fitted pair potentials. According to our analysis, the origin of the (2 × 1) reconstruction of calcite(104) is purely thermodynamic when accounting for all relevant physical interactions in DFT. We also excluded that impurities and/or defects cause the reconstruction by analyzing calcite(104) slabs with cation subsurface vacancies as well as Mg and Sr dopants at different concentrations (see Table 1), thereby rebutting previous suggestions for surface reconstructions of calcite(104).^26,27^ Overall, the presence of these dopants and defects did not play any role in stabilizing the (2 × 1) reconstruction, with Mg even destabilizing it. In order to validate this predicted model, we now compare it in detail to experimental NC-AFM image data.

**Figure 2:**
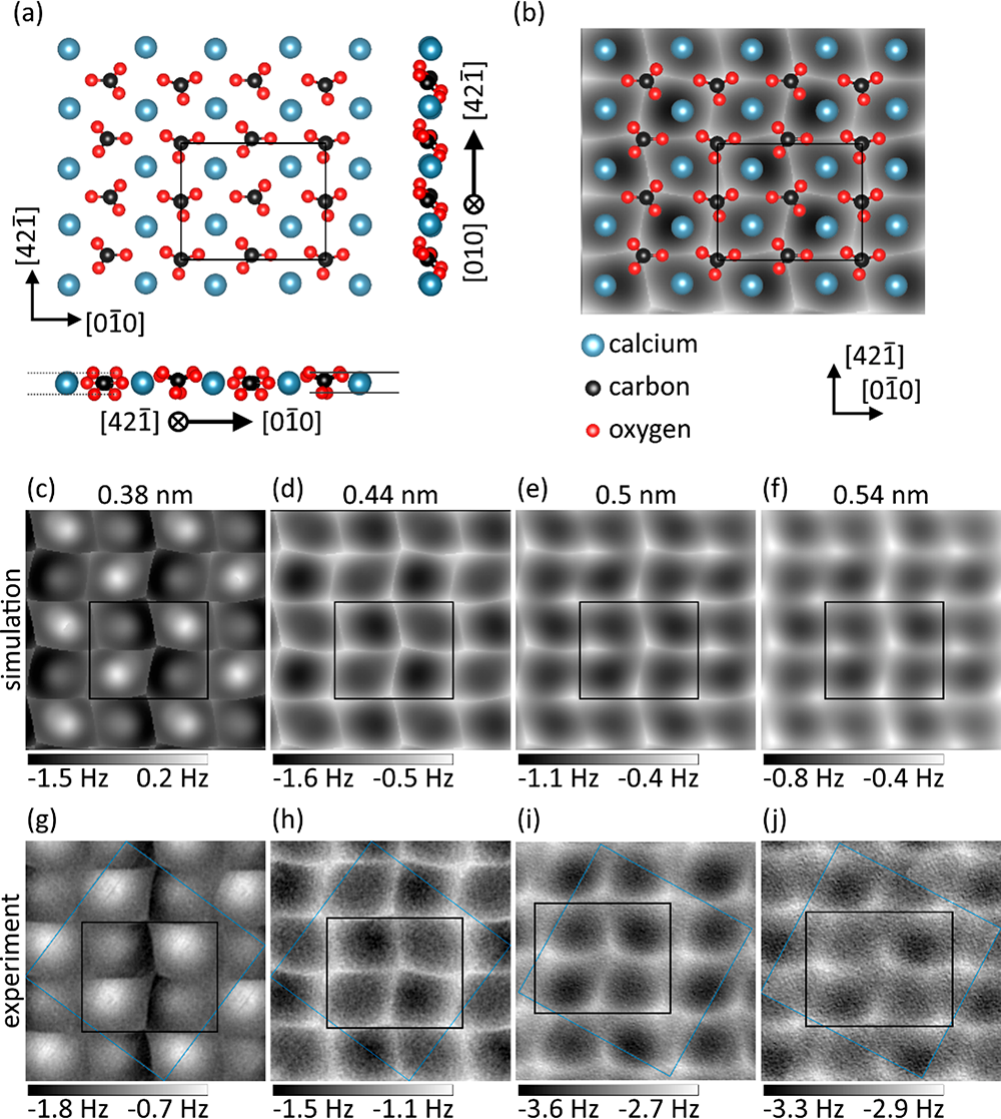
Microscopic geometry and high-resolution NC-AFM imaging of the (2 × 1) reconstructed calcite(104) surface. (a) Microscopic surface structure as determined by DFT geometry relaxation (only top layer shown). The reconstruction is predominantly confined to the first layer; lower-lying layers closely resemble the bulk structure. Dashed (solid) horizontal lines on the left (right) side in the side projection mark the z-planes of the highest and lowest oxygen atoms of the unreconstructed (reconstructed) carbonate groups at about +70 pm and −80 pm (±70 pm) above and below the Ca plane. While the top oxygen atoms are at the same height for the unreconstructed row, the oxygen atoms of reconstructed row have a height difference of about 20 pm. (b) Simulated NC-AFM frequency shift image with overlaid atomic model. Distance-dependent contrast formation of the (2 × 1) reconstructed surface from (c–f) simulated image data and (g–j) high-resolution frequency shift data acquired with CO-terminated tips at 5 K (unit cell average, crystallographic unit cell marked in black, averaging cell marked in blue, data periodically extended beyond averaging cell; a total of 10, 17, 11, and 11 unit cells from four images were averaged for panels g, h, i, and j, respectively). Data were acquired with two CO tips (g/h and i/j each with identical tip). Unit cell sizes (black rectangles): 1.0 × 0.81 nm^2^. PPM parameters: *A* = 0.35 nm (zero-peak; same contrast for *A* = 0.75 nm, data not shown). Experimental parameters: (g and h) *A* = 0.77 nm; (i and j) *A* = 0.38 nm. All features are rather robust against PPM parameter choice (see Figure S10).

**Table 1:** Energetic Differences in Simulations between a (1 × 1) and (2 × 1) Reconstructed Calcite(104) Slab Determined for Mg and Sr Surface Dopants at a Concentration of 1.6% (Each Dopant Substitutes One Ca Atom in the Supercell) and a Cation Subsurface Vacancy^a^

system comparison ((2 × 1) – (1 × 1))	energy difference (eV)
CaCO_3_	–0.21^b^ (−0.15^c^, −0.13^d^, +0.02^e^)
CaCO_3_—Mg	0.01
CaCO_3_—Sr	–0.17
CaCO_3_—vacancy	–0.08

a The dopants Mg and Sr were chosen as they were found in trace amounts in the calcite crystals used for the experiments (Mg (0.05–0.5) %, Sr (0.003–0.02) %, Mn ≤ 0.005%). Overall, the presence of these dopants and defects did not play a significant role in stabilizing the (2 × 1) reconstruction, and Mg actually destabilized it. For the comparison of the ideal (1 × 1) and (2 × 1) surfaces in the first row, we also give the values for several DFT functionals:

b PBE^35^ with the Tkatchenko–Scheffler method with iterative Hirshfeld partitioning.^36^

c DFT-D3 with Becke–Johnson damping.^37,38^

d HSE06^39^ and the Tkatchenko–Scheffler method with iterative Hirshfeld partitioning.

e PBE with no van der Waals interaction. For each functional, the cell parameters were fully optimized before relaxing the surface structure.

NC-AFM images simulated at four different tip–sample distances are reproduced in Figure 2c–f. The simulations use the probe particle model (PPM),^30^ which takes electrostatic forces, London dispersion interactions, and Pauli repulsion together with the flexibility of the CO molecule attached to the tip into account (see the Supporting Information for further details). For all calculations, an oscillation amplitude of *A* = 0.35 nm is used and the tip–sample distances are referred to the average top-layer calcium position along *z*. A number of features specific to imaging the (2 × 1) reconstructed calcite(104) surface with a CO tip can be unravelled at all tip–sample distances in the simulated images: (i) The lattice is formed by bright lines running in  and  directions; lines running along the  direction can have a different width and brightness following the (2 × 1) reconstruction. (ii) The lines along the  direction express a zigzag pattern. (iii) The lines along  are slightly bent in the  direction. (iv) Diagonally adjacent dark pores have a similar appearance producing a checkerboard-like pattern, whereby the position of the dark pores within the unit cell changes with tip–sample distance. (v) The axes of glide reflection run along  and are centered on the bright vertical lines. These features are all found in experimental NC-AFM image data, see Figure 2g–j, where unit cell averaged data are shown. In particular, (i) the bright lines including (ii) the zigzag and (iii) the slight bent give an excellent match. Furthermore, (iv) the checkerboard-like pattern is clearly seen and sharpened at *z* = 0.38 nm, see Figure 2c,g. There are two additional effects in the imaging that depend on the tip–sample distance. First, the checkerboard-like pattern changes the orientation: While the upper right and lower left Ca atoms within the illustrated unit cell interact at large tip–sample distances (see Figure 2e,f,i,j) more attractive with the CO tip than the other two calcium atoms, this order is reversed at close tip–sample distances (Figure 2d,h) where instead the upper left and lower right calcium atoms express the stronger attractive interaction. Second, a rather abrupt change in the imaging is observed at very close tip–sample distances (Figure 2c,g), where strong repulsive interactions and flexing of the tip molecule lead to an inverted appearance of the calcite(104) surface. Thus, the excellent match between the simulated and experimental data clearly validates the DFT-derived geometry of the reconstructed surface.

Carbon monoxide (CO) molecules deliver an excellent probe molecule for investigating effects of the reconstruction and surface symmetry properties on molecular adsorption due to the exclusive adsorption of CO at the calcium ion site.^31^ Principally, there are four adsorption sites I, I^g^, II, and II^g^ (see markers in Figure 3a) present within the (2 × 1) unit cell; two of them are linked by the glide plane symmetry, and two of them are fundamentally different due to the (2 × 1) reconstruction. Constant-height data in Figure 3a present four single CO molecules as bright features positioned at the dark pores of the calcite lattice image; image subparts presenting CO molecules at positions I and II are extracted and reproduced with the  direction pointing upward in Figure 3c,e (see Figure S5d for confirmation of the glide plane symmetry element for I and I^g^). Single CO molecules are imaged as a bright repulsive center (marked by an orange dot) with sharp filaments connecting with the surrounding calcite lattice image (ends marked by short orange lines), located mainly at the connecting lines between the CO molecule and each of the surrounding carbonate groups. By inspecting the fine substructure of the CO molecule in the constant-height images, it is clearly apparent that the substructure of the two CO molecules in Figure 3c,e is markedly different: the number and exact orientation of the sharp filaments differ between the CO molecules adsorbed at sites I and II. Additionally, the respective intensity of the two molecular images is different, highlighting a less repulsive interaction with the CO at site II compared to site I. As it is known that the imaging contrast with a CO tip is critically sensitive to the atomic positions and molecular geometry,^30^ this observation clearly demonstrates the influence of the (2 × 1) reconstruction on the adsorbed species and allows in this case the identification of CO types I and II.

**Figure 3:**
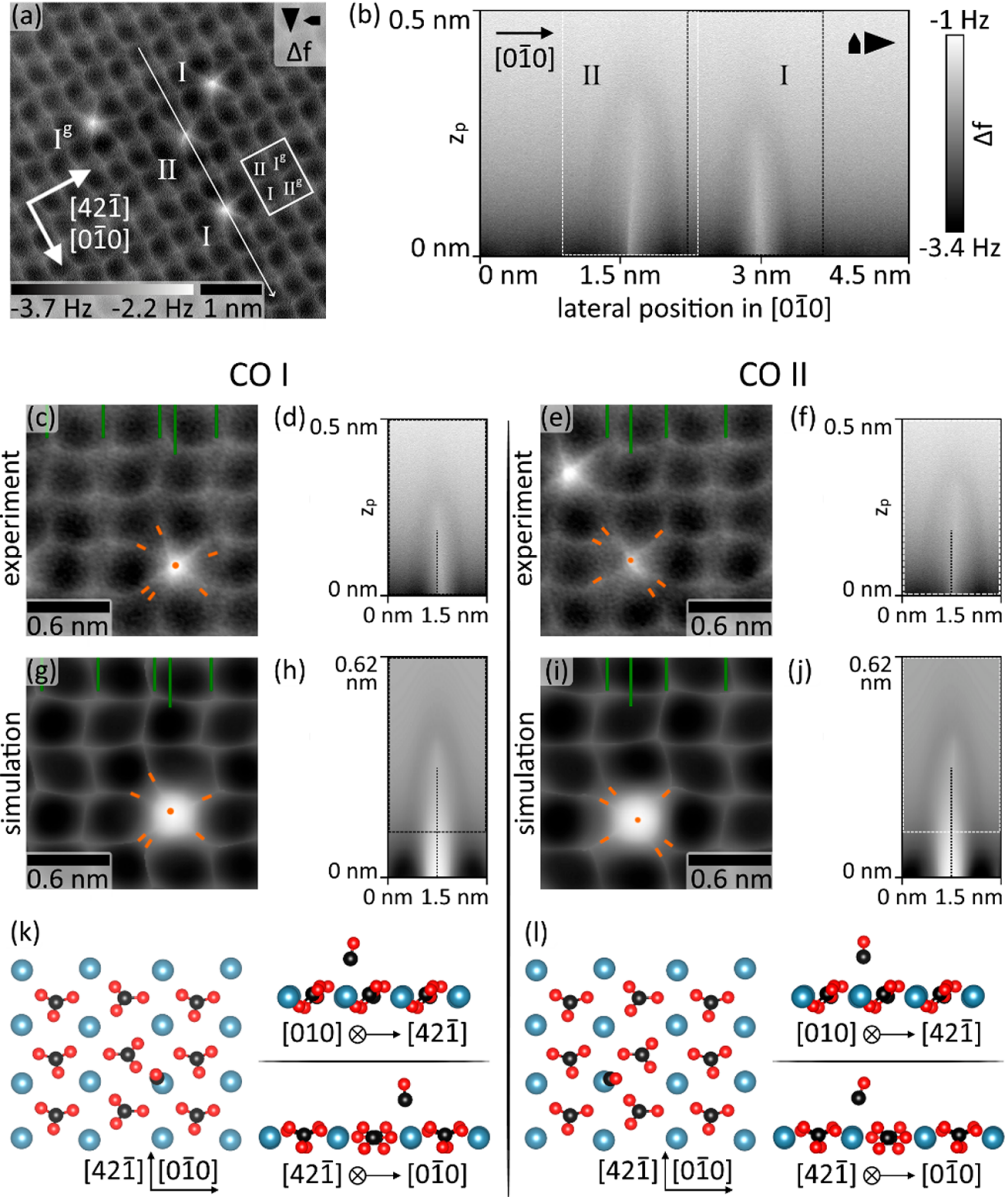
Two different adsorption sites of CO inside the (2 × 1) reconstructed unit cell. (a) Constant-height frequency-shift NC-AFM image acquired with a CO-functionalized tip (*A* = 0.38 nm) of CO/calcite(104)–(2 × 1). Four CO molecules are identified as bright protrusions that replace a dark feature at the Ca site. (b) XZ frequency-shift slice data acquired along the long white line with arrow in panel a, crossing molecules of type I and II along the  direction (slice data acquired along the  direction is shown in Figure S7). Both CO molecules are apparent as bright (repulsive) features with a dark (attractive) surrounding. (c–l) Analysis of two different CO adsorption geometries for (c, d, g, h, and k) CO type I and (e, f, i, j, and l) CO type II. (c and e) High-resolution experimental constant-height images (extracted from panel a) and (d and f) experimental slice data (extracted from panel b as marked) are compared with the corresponding PPM simulations (g–j). Green lines act as reference markers for the carbonate rows and the CO center positions in the experimental data. (k and l) Geometries for the calculated models showing the closest match between theory and experiment. Top- and side views reveal only minor modification of the calcite(104)–(2 × 1) surface geometry due to the CO adsorption. Calculated CO adsorption energies: (k) −0.38 eV and (l) −0.30 eV.

The difference between CO at sites I and II is further evidenced when imaging CO/calcite(104) at moderate distances in topography mode (see Figure S5 where a difference in the imaged size of the two CO types is visible), by investigating neighboring dimers containing type I and II CO (see Figure S6 where some of the neighboring dimers express an apparent bond in NC-AFM imaging) and by systematically performing distance-dependent measurements along the  direction as shown in Figure 3b. The vertical interaction reproduced by the slice data in Figure 3b reveals a weak attractive regime around a strong and sharp repulsive center for each CO molecule, in agreement with previous measurements of CO/Cu(111).^33,40^ Specifically, the CO molecule at site II appears higher than at site I, in agreement with the apparent larger size of CO molecules at site II in topography data (see Figure S5). Furthermore, the slice data show a tilt of the repulsive core with respect to the surface normal. Most importantly, the orientation of this tilt is different: the CO molecule at site I (site II) is tilted to the left (right) along the  direction. As the tilt orientation differs within the same data set, a measurement artifact due to a tilted CO tip can be excluded. Thus, the experimental data strongly supports the presence of two molecular adsorption geometries, namely, CO types I and II, generated by the (2 × 1) reconstruction of calcite(104).

Several starting geometries for CO/calcite(104)–(2 × 1) were optimized by DFT. The results can generally be divided into two categories differing in their total energy by up to nearly 0.1 eV, corresponding to the different CO types I (also type I^g^, with lower total energy, see Figure S8) and II (also type II^g^, with higher total energy, see Figure S9). NC-AFM images were simulated for all cases and compared with the experimental constant-height image and slice data; the best match is shown in Figure 3g–j for the two geometries reproduced in Figure 3k,l. Experimentally imaged features, especially the repulsive fine structures, are excellently reproduced in the simulated image data in Figure 3, giving confidence in the DFT models and delivering clear evidence for an influence of the (2 × 1) reconstruction on adsorbed CO molecules.

In conclusion, we have shown that the success of high-resolution AFM with functionalized tips in imaging molecular processes,^28,41^ thin-film systems,^42,43^ or oxide surfaces^44^ can be brought to resolution of the structure of insulating bulk minerals. Our experimental data acquired at 5 K with well-characterized and functionalized tips consistently express a (2 × 1) reconstruction of a *pg* surface with the axes of glide reflection located on the rows of the carbonate groups in the  direction. Combined with results from the literature where a (2 × 1) reconstruction has been observed at room temperature,^13,18^ it appears the (2 × 1) reconstruction exists over a large temperature range. Most importantly, the surface reconstruction has a clear impact on adsorbent properties, influencing their adsorption geometry and energy. In future studies, these differences in the adsorption position have to be investigated for the plethora of organic and inorganic molecules that are known to interact with calcite in geochemical or biological contexts in both hydrated and pristine forms. Therefore, this finding is most critical for future studies where physical processes on calcite(104)–(2 × 1) *pg* can be influenced by the surface microscopic structure.

## Supporting Information Available

Geometrical structure of calcite(104)–(2 × 1) (CIF)
Materials and Methods: Sample preparation, STM and NC-AFM experiments, description of the algorithmic symmetry test, DFT calculations, and PPM image calculations including illustration of the two-atom probe particle model (Figure S1); extended details for the algorithmic symmetry test performed in Figure 1 (Figures S2) and examples of applying the symmetry test to data acquired with sharp (Figure S3) and unknown (Figure S4) tips; further experimental and theoretical results describing the CO adsorption: identification of two species (Figure S5), NC-AFM imaging of CO dimers (Figure S6), slice data acquired along the [421̅] direction (Figure S7), as well as DFT models for CO types I/I^g^ (Figure S8) and II/II^g^ (Figure S9); parameter analysis for PPM calculations (Figure S10) (PDF)

## Supplementary Material

jz2c03243_si_001.cif
