# Supplementary material for: Differences in Molecular Adsorption Emanating from the (2 × 1) Reconstruction of Calcite(104)
Source: J Phys Chem Lett. 2023 Feb 16;14(7):1983–9. doi: 10.1021/acs.jpclett.2c03243 (PMC10100545; doi:10.1021/acs.jpclett.2c03243)
Supplement: Supplementary file 2 — jz2c03243_si_002.pdf [file jz2c03243_si_002.pdf]

Supplemental Materials

Differences in Molecular Adsorption Emanating from the (2×1) Reconstruction of Calcite(104)

Jonas Heggemann[1], Yashasvi S. Ranawat[2], Ondřej Krejčí[2], Adam S. Foster[2,3], Philipp Rahe[1]**

[1]Fachbereich Physik, Universität Osnabrück; 49076 Osnabrück, Germany.

[2]Department of Applied Physics, Aalto University; Helsinki FI-00076, Finland.

[3]Nano Life Science Institute (WPI-NanoLSI), Kanazawa University; Kanazawa 920-1192, Japan.

*Corresponding authors. Email: adam.foster@aalto.fi (ASF), prahe@uos.de (PR).

Materials and Methods

Sample preparation

Sample preparation and NC-AFM experiments were performed under ultra-high vacuum conditions ($p < 2\times10^{-10}$ mbar). A double sample holder[1] supporting both a Ag(111) metal sample for tip preparation and the calcite(104) sample for NC-AFM experiments was used. Metal surfaces were prepared by common sputter (Ar^+, $E = 1$ keV, $p_{Ar} \sim 1.5 \times 10^{-6}$mbar) and anneal (15 min, about 300°C) cycles. Calcite crystals (Korth Kristalle, Altenholz, Germany) were cleaved[2] *in-situ* parallel to a (104) surface after preparing the metal samples, followed by heating the crystal for 1.5 h at about 170°C to remove residual charges[3]. The crystals were optically transparent, trace amounts of Mg and Sr were found from ICP-OES measurements. CO was dosed directly into the cold scanhead ($T_{sample} < 7$ K while opening the shields) for 120 s ($p_{CO} \sim 3 \times 10^{-8}$ mbar). The orientation of the $[42\bar{1}]$ direction was determined *ex-situ* by an optical procedure after the NC-AFM experiments[4]: The split between the ordinary and extraordinary ray is pointing along the $[42\bar{1}]$ direction.

STM and NC-AFM experiments

STM and NC-AFM experiments were performed using an LT qPlus gen.III instrument (ScientaOmicron, Taunusstein, Germany) operated by a MATRIX controller and with an atom-tracking system for drift compensation[5]. Electrochemically etched tungsten tips attached to qPlus sensors[6] as supplied by the manufacturer were used. Tips were prepared on metal samples by common STM strategies and CO pick-up was performed using z-ramps in STM mode. NC-AFM

was performed in the frequency-modulation constant-amplitude mode[7]. Oscillation amplitudes (zero-peak) of 0.3 nm to 1.0 nm were used and calibrated with the normalized time-averaged tunneling current method[8]. The fast and slow scan directions are indicated by an arrow and a triangle, respectively, in the experimental images. During the measurements on calcite(104), a bias voltage was applied to reduce electrostatic background forces (Fig. 1: U_{bias} = 10 V; Fig. 2(g,h): U_{bias} = –3 V; Fig. 2(i,k): U_{bias} = 10 V). Additionally, residual drift was corrected by comparing pairs of up and down images[9] and accordingly shearing the image data. Slice data in Fig. 3(b) (supplementary materials Fig. S7(f,g)) consist of a total of 375 (200) Δf(z) curves. Image data were processed using Gwyddion[10].

Algorithmic symmetry test

The algorithmic symmetry test quantifies the presence of a glide plane reflection symmetry element in NC-AFM images. The quantification procedure compares the values $y(i,j)$ at all measurement positions $[i,j]$ within a single (2×1) unit cell with the values $y(i_{pg}, j_{pg})$ extracted from the corresponding g-equivalent positions $[i_{pg}, j_{pg}]$ in the image data. These two coordinate sets are linked by the reflection and translation operation of the g symmetry element, namely by $[i_{pg}, j_{pg}] = [i,j] + 2 * m * \frac{\vec{e}_{[0\bar{1}0]}}{|\vec{e}_{[0\bar{1}0]}|} + \frac{\vec{e}_{[42\bar{1}]}}{2}$, with $m = \frac{|([i,j] - \vec{s}_X) \times \vec{e}_{[42\bar{1}]}|}{|\vec{e}_{[42\bar{1}]}|}$ defining the shortest distance of $[i,j]$ to the axis of glide reflection. The vector $\vec{e}_{[42\bar{1}]}$ ($\vec{e}_{[0\bar{1}0]}$) is a vector in $[42\bar{1}]$ ($[0\bar{1}0]$)-direction with length of one unit cell in $[42\bar{1}]$ ($[0\bar{1}0]$)-direction, and $\vec{s}_X$ is a support vector to an arbitrary point on the axis of glide reflection. Here we use the crossing point between the axis and the lower boundary of the black rectangle. Note that $\vec{e}_{[42\bar{1}]}$ and $\vec{e}_{[0\bar{1}0]}$ are defined in a

Cartesian coordinate system[4], while the $[42\bar{1}]$ and $[0\bar{1}0]$ directions relate to the hexagonal crystal system.

The numerical difference of the values at the original and *g*-equivalent positions, evaluated for each measurement point $[i,j]$ within the (2×1) unit cell, is given by $\Delta^{pg,X}(i,j) = y(i,j) - y(i^X_{pg}, j^X_{pg})$ and allows the calculation of the total RMS deviation $\Delta^{pg,X} = \frac{1}{N}\sqrt{\sum_{i,j}\left(\Delta^{pg,X}_{i,j}\right)^2}$, whereby *N* represents the total number of measurement points $[i,j]$ inside the (2×1) unit cell. To account for imperfections in positioning the axes of glide reflection in the experimental image, thereby ensuring the best positioning of these axes, the *g* translation vectors are shifted for a series of systematic relative shifts $[i^X_{pg}, j^X_{pg}] \to [i^X_{pg} + m, j^X_{pg} + n]$, effectively translating the *g*-processed unit cell systematically by *(m, n)* pixel along the main surface directions. The smallest RMS deviation $\Delta^{pg,X}_{min} = min_{m,n}\Delta^{pg,X}_{m,n} = min_{m,n}\frac{1}{N}\sqrt{\sum_{i,j}\left(y(i,j) - y(i^X_{pg} + m, j^X_{pg} + n)\right)^2}$ then represents the optimum overlap of the reference and *g*-equivalent unit cell data. For an ideal and noise-free measurement, the deviation $\Delta^{pg,X}_{min}$ for the optimum overlap would be zero. For real experimental data, however, a finite deviation will always be present due to noise in the measurement data[11].

Consequently, in order to assess the magnitude of the deviation resulting from the *g* symmetry analysis and to compare it with the noise background, the RMS deviation Δ^t is additionally calculated for a unit cell translated by a multiple of the lattice vectors. In particular, the measurement values $y(i_t, j_t)$ at the data points $[i_t, j_t] = [i,j] + \vec{a}$, with $\vec{a}$ being an integer multiple of the lattice vector along one main surface direction, are used to calculate

$\Delta^t_{min} = min_{m,n}\Delta^t_{m,n} = min_{m,n}\frac{1}{N}\sqrt{\sum_{i,j}\left(y(i,j) - y(i_t + m, j_t + n)\right)^2}$. In full analogy to the formulae for $\Delta^{pg,X}_{min}$, the minimum value Δ^t_{min} is used as a lower limit for the g symmetry check confidence by calculating the relative deviation $\beta_X = \left(\Delta^{pg,X}_{min} - \Delta^t_{min}\right)/\Delta^t_{min}$. Example data for $\Delta^{pg,X}_{m,n}$ and $\Delta^t_{m,n}$ are shown in Fig. S1.

DFT calculations

All first-principles calculations in this work were performed using the periodic plane-wave basis VASP code[12-13] implementing the spin-polarized Density Functional Theory. To accurately include van der Waals interactions for this system, we used the Tkatchenko−Scheffler method with iterative Hirshfeld partitioning[14], shown to be best suited for ionic systems[15-16]. Various other van der Waals[17-18] and hybrid[19] functionals were tested and the (2×1) reconstruction was consistently predicted to have a lower energy that the (1×1) surface (see Table 1). Calculations with no van der Waals predicted that the (1×1) surface was slightly favoured. Projected augmented wave potentials were used to describe the core electrons[20] with a kinetic energy cutoff of 500 eV (with PREC = accurate). Systematic k-point convergence was checked for all systems with sampling chosen according to the system size. This approach converged the total energy of all the systems to the order of 1 meV. The properties of the bulk calcite were carefully checked within this methodology, and excellent agreement was achieved with experiments. For calculations of the surface, we used a $4 \times 4 \times 4$ supercell (320 atoms total), a vacuum gap of at least 1.5 nm and a $5 \times 5 \times 1$ k-point grid. The upper two layers of calcite were allowed to relax to a force of less than 0.001 eV/Å. Allowing all atoms in the slab to relax made no significant difference to the stability

of the (2×1) reconstruction. Energy differences refer to comparisons between equivalent supercells.

PPM image calculations

The probe particle model (PPM)[21-22] was used for simulating the high-resolution AFM images, using the two-atom flexible CO tip-apex configuration[23]. An illustration of the method is shown in Fig. S1. The parameters of the tip geometry, its stiffness values, and electrostatic properties were fitted by simulating interactions over a pentacene molecule, using DFT[23]. The electrostatic interactions between atoms of the tip and sample were taken into account using the Hartree potential of the surface[22]. For the remaining interactions, the Lennard−Jones equilibrium distance parameters were fitted for each individual atom of the $CaCO_3$ sample in the top-most layer of the surface using a recent probe-particle-model fitting procedure [https://github.com/ProkopHapala/ProbeParticleModel/tree/OpenCL_py3], as used on other ionic systems[24-25].

To establish the consistency of our simulated images, a wide range of PPM images were computed by systematically varying the radial-stiffness, z-positions, tilt-angle of C and O in the molecule, the separation of metallic atoms in the tip, and the stiffness of the tip-apex by ±10%. Moreover, the charges on the atoms were varied by ±200%, to detect the correlations in the simulated images with the parameters. The results of this extended scan can be found in supplementary materials Fig. S10.

Note that the tip-bound CO is allowed to fully relax within the PPM while the surface (including adsorbed CO) is constrained. This is the most probable explanation for any small differences between the simulated and experimental images.

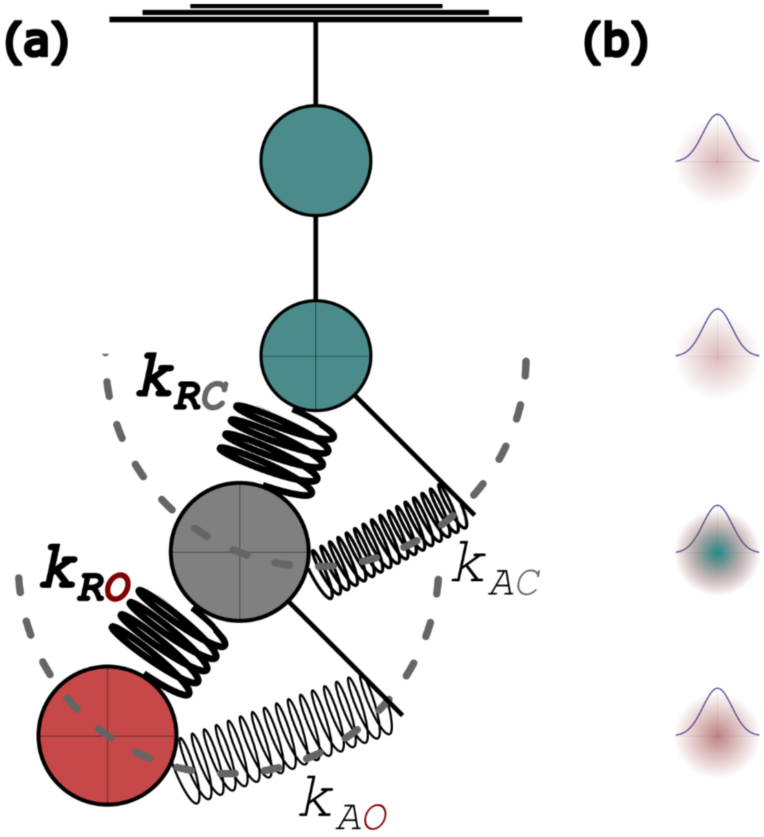

Figure S1:

Illustration of the two-atom Probe Particle Model – a molecular mechanics model of the CO functionalized tip allowing for a full relaxation of carbon (depicted in grey) and oxygen (red) atoms in the force-fields of the flexible tip and fixed sample atoms[21]. (a) The tip force-field is composed of a stiff radial spring (k_R) and soft angle springs (k_A), representing the flexibility of the attached CO. The tip-sample interaction consists of pair-wise Lennard−Jones potentials, representing London dispersion and Pauli repulsion interactions. Additionally, electrostatic forces are computed using the Hartree potential from DFT[22] calculations of the surface and Gaussian charge clouds centred on each of the tip atoms as sketched in (b). The four charges on the minimalistic Cu_2−C−O tip model were fitted from DFT calculations[23]. The color and intensity illustrate the sign and magnitude of the charge (green−blue is positive, red is negative).

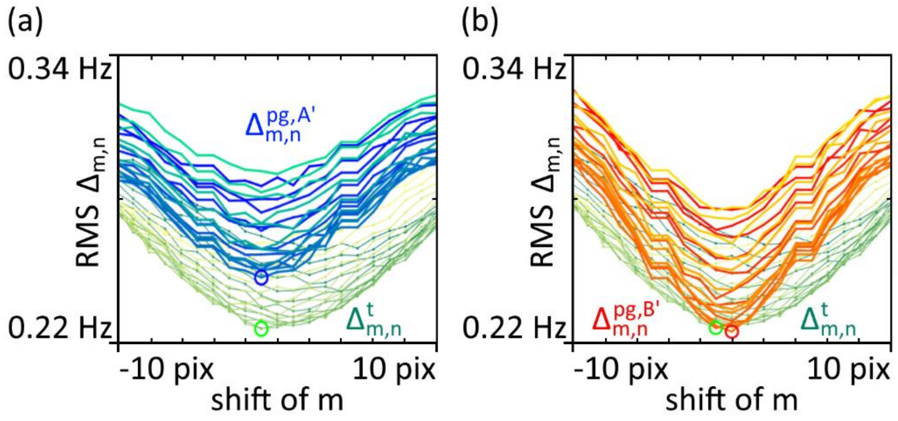

Figure S2:

Algorithmic symmetry test: $\Delta^{pg,X}_{m,n}$ and $\Delta^{t}_{m,n}$ corresponding to the data shown in Fig. 1(a-c). The RMS deviation for the axes of glide reflection positioned on (a) axis A' and on (b) axis B' are shown. While the shift of the axes along m are directly plotted, each curve represents a different n.

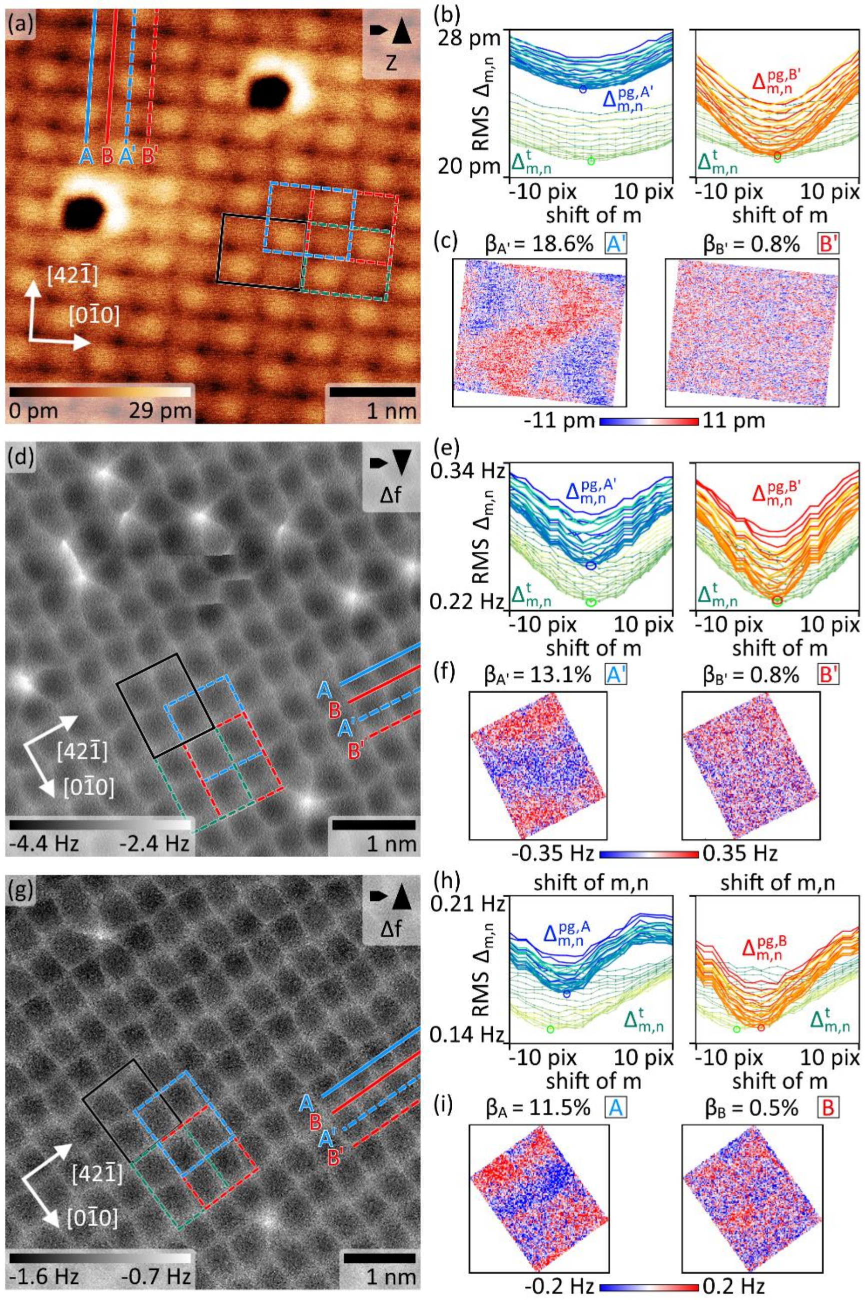

Figure S3:

Algorithmic symmetry test applied to measurement data acquired with (a-c) sharp and (d-i) CO terminated tips. (a) High-resolution NC-AFM image acquired in topography mode on calcite(104) at 5 K ($\Delta f = -1.67$Hz and $U_{bias} = 6$ V). (b,c) Results of the algorithmic symmetry test using data marked by the black rectangle in (a). (d) Constant-height frequency-shift image acquired with a CO-functionalised tip ($U_{bias} = 10$ V). (e,f) Algorithmic symmetry test using the unit cell marked in (d) by a black rectangle. (g) Constant-height frequency-shift image acquired with a different CO-functionalised tip ($U_{bias} = -3$ V). (e,f) Algorithmic symmetry test using the unit cell marked in (g) by a black rectangle.

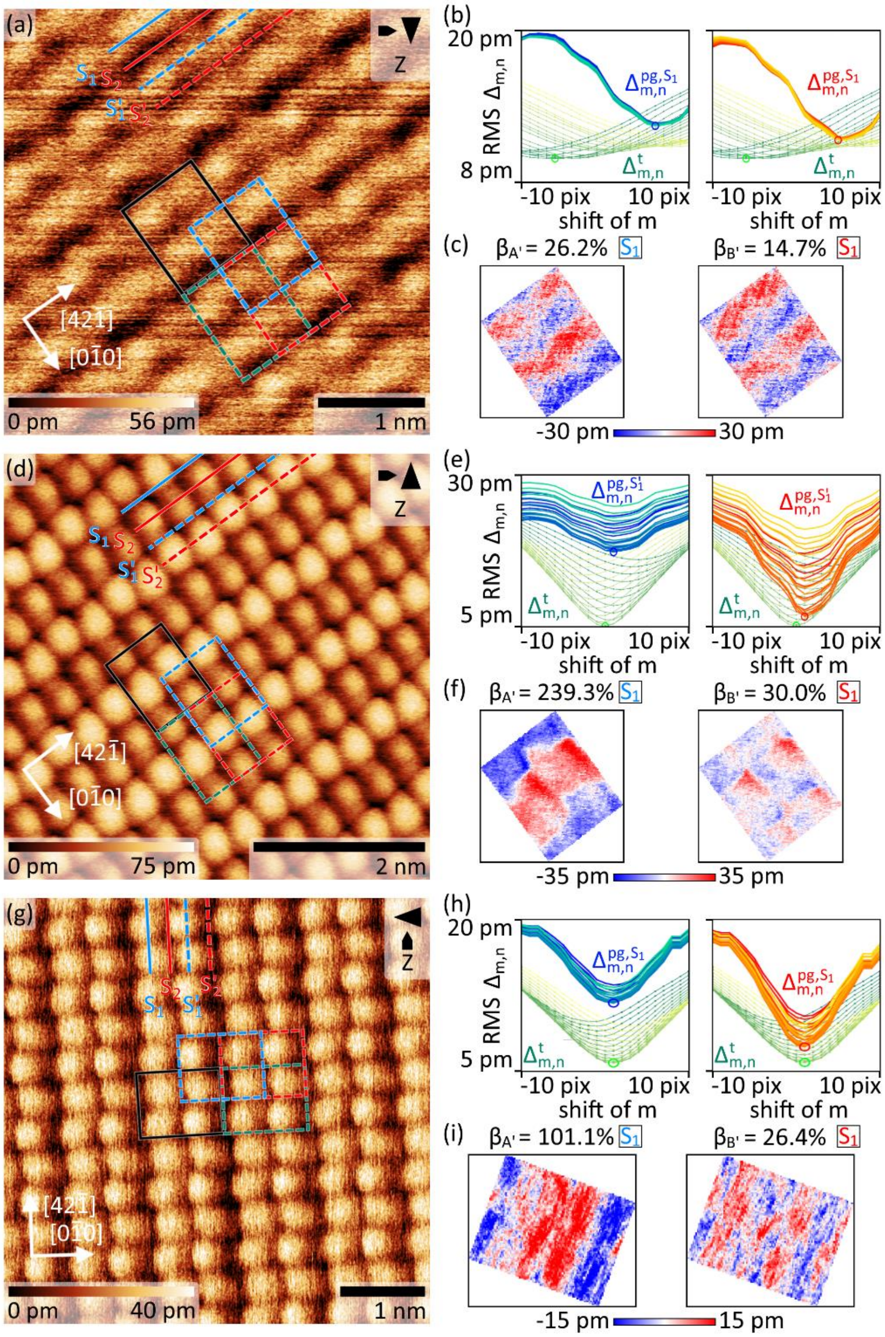

Figure S4:

Algorithmic symmetry test applied to NC-AFM data acquired with unknown tips, all violating the *g* symmetry element. None of the tips were terminated with a CO molecule. (a) Exemplary data expressing the row-pairing reconstruction (Δf = –2.8 Hz and U_{bias} = 0 V). (b,c) Local deviation within a (2×1) unit cell with axes of glide reflection S_1 and S_2 positioned on bright and dark rows, respectively. (d) High-resolution image with sharp contrast achieved with an unknown tip. (Δf = –3.3 Hz and U_{bias} = 1 V). (e,f) Local deviation resulting from the symmetry test. (g) Image of calcite(104) with violated *g* symmetry element (Δf = –1.5 Hz and U_{bias} = 9 V). (h,i) Local deviation calculated by the symmetry test.

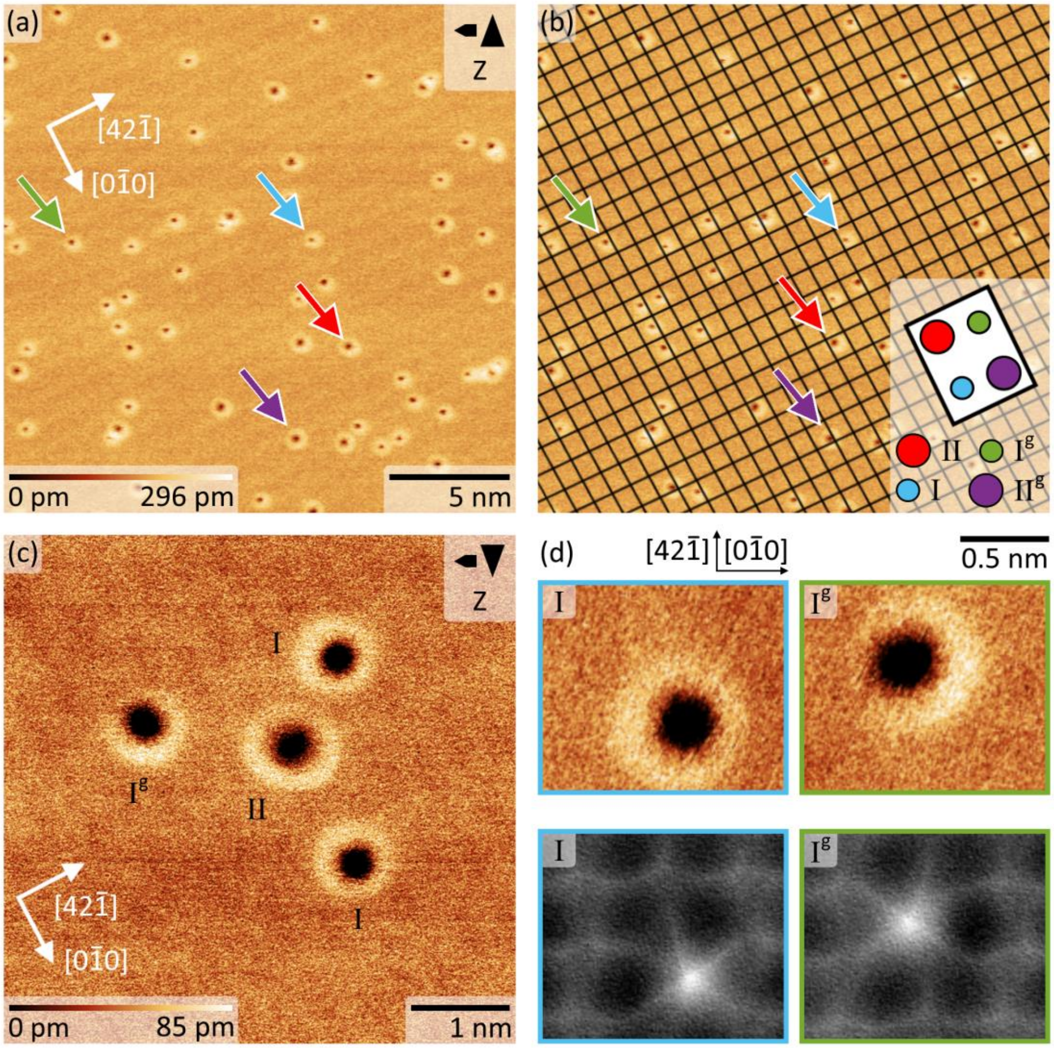

Figure S5:

Properties of the CO/calcite(104) adsorption positions. (a) NC-AFM topography image of CO/calcite(104) (Δf = –0.9 Hz and U_{bias} = 10 V) at moderate tip–sample distance. Single CO molecules are apparent as dark features with a bright rim and the underlying calcite lattice is faintly visible in the form or stripes along the [42$\bar{1}$] direction. Coloured arrows mark different CO adsorption sites. (b) Same data with a carefully aligned (2 × 1) grid as overlay. The grid lines are equidistant and represent the lattice of a (2 × 1) reconstructed calcite surface with a periodicity of 1 nm in [0$\bar{1}$0] direction and 0.81 nm in [42$\bar{1}$] direction. The grid is slightly sheared to take the imperfections of the piezo scanner into account. The lines of this grid are aligned by using 2D Fourier filtering and fine-adjusted by matching all CO positions to one of the four respective positions within the (2 × 1) unit cell sketched in the inset. (c) Topography image (Δf = –2.1 Hz and U_{bias} = 10 V) of four CO molecules on the calcite(104) surface. (d) Data extracted around two different CO molecules from (c) with the [42$\bar{1}$] direction pointing upwards. Upper row: topography data. Lower row: constant-height frequency-shift data. Images for I and I^g are related by the glide plane reflection element, highlighting that the CO molecules follow this surface symmetry property of the surface.

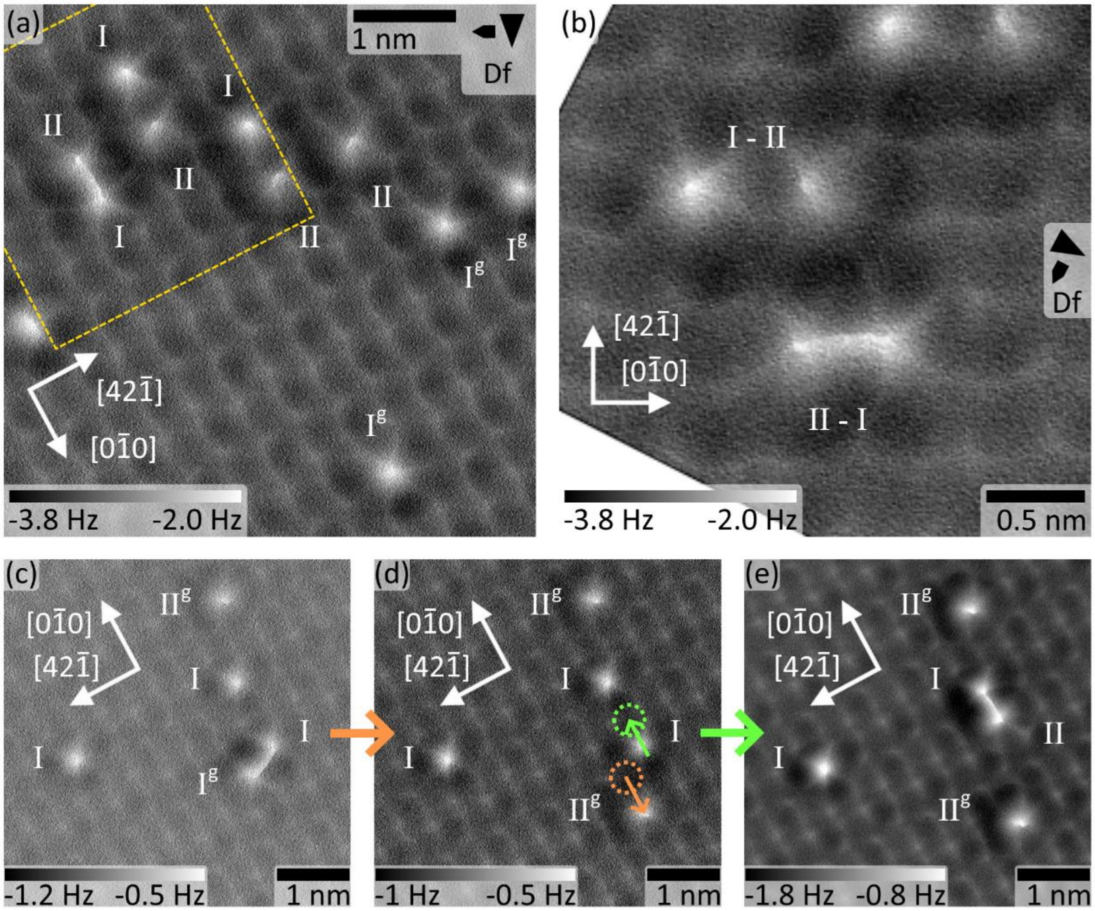

Figure S6:

NC-AFM imaging of CO dimers. (a) Constant-height frequency-shift image, CO molecules are apparent as single bright features, the calcite(104) lattice is imaged as a bright lattice (see main text for further details). (b) Excerpt from image (a) as marked by a yellow dashed box. An apparent bond is observed between the II–I CO dimer (bottom), while no line is visible for a I–II CO dimer (top), both aligned along the $[0\bar{1}0]$ direction. (c–e) Dimer formation and dispersion by lateral manipulation of the CO molecules during scanning. Manipulation occurs while scanning the CO/calcite(104) surface in constant-height mode, the original and new positions are marked by dashed circles and arrows, respectively. There is now a consensus that NC-AFM with functionalised tips may indicate bonds between non-bonded features and that the appearance of these bonds is rather an effect of the proximity of two atoms than a result of electron density increase or real bonding[21, 26]. We therefore conclude that the tilt of the CO molecules is the origin of the apparent bond between a II–I CO dimer as this tilt brings the top oxygen atoms closer towards each other for a II–I dimer, see slice data in Fig. 3(b). In contrast, the CO molecules are tilted away from each other in a I–II CO dimer, in agreement with a separate imaging of the two molecules.

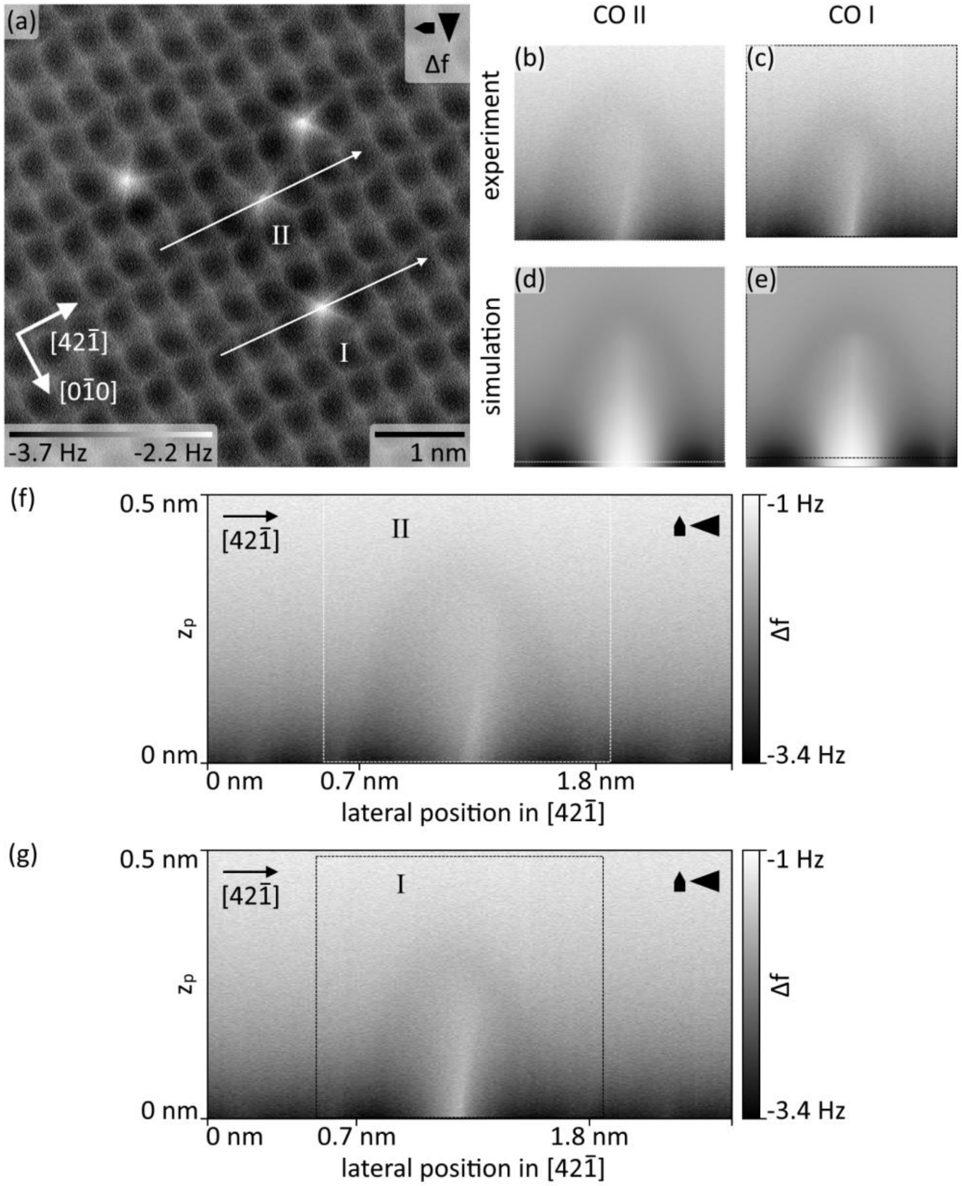

Figure S7:

Slice data along the $[42\bar{1}]$ direction across both CO types. (a) Image reproduced from Fig. 3(a) with the positions of the two slice data acquired along the $[42\bar{1}]$ direction marked. (f) Slice data represented along the $[42\bar{1}]$ direction across a CO molecule of type II. (g) Slice data represented along the $[42\bar{1}]$ direction across a CO molecule of type I. Slice data was acquired along the $[\overline{42}1]$ direction, for consistency data are shown with the lateral axes flipped horizontally. (b–e) Comparison between (b,c) experimental and (d,e) simulated slice data. Model *B* is shown in (d) and model *A* is shown in (e). See Fig. 3 in the main text for further details.

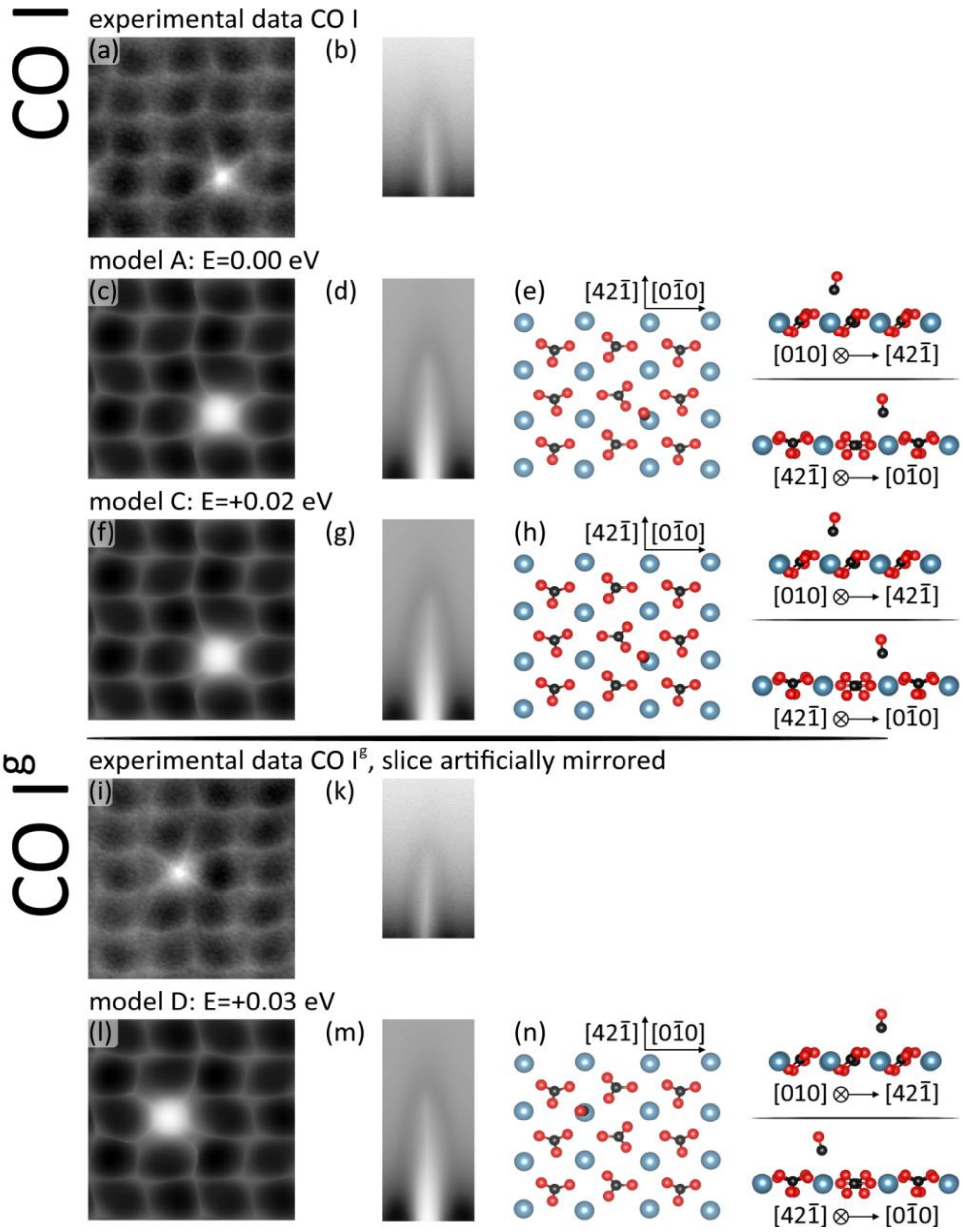

Figure S8:

Comparison of various calculated models for the CO adsorption types I and I^g, extending the results in Figure 3 of the main text. (a) Experimental XY image and (b) experimental XZ scan of the CO type I. (c,d) NC-AFM image simulation and (e) DFT geometry for the best-matching model *A* (also shown in Fig. 3). (f,g) NC-AFM image simulation and (h) DFT geometry for model *C* with slightly higher energy and reduced match between experimental and simulated NC-AFM data. (i) Experimental XY image of CO adsorption type I^g and (k) mirrored experimental XZ scan from (b) to represent the CO I^g symmetry. (l,m) NC-AFM image simulation and (n) DFT geometry for model *D* with also slightly higher energy and reduced match between experimental and simulated NC-AFM data.

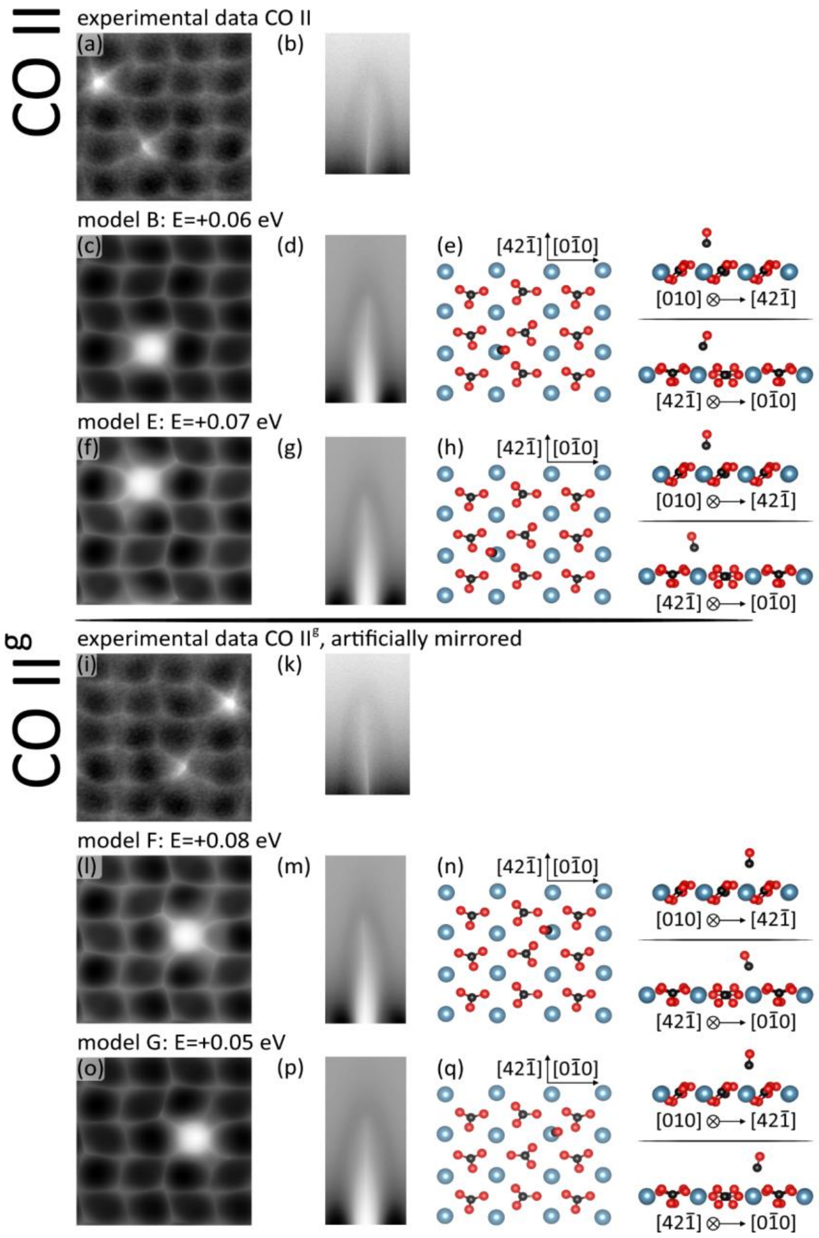

Figure S9:

Comparison of various calculated models for the CO adsorption types II and II^g, extending the results in Figure 3 of the main text. (a) Experimental XY image and (b) experimental XZ scan of the CO type II. (c,d) NC-AFM image simulation and (e) DFT geometry for the best-matching model *B* (shown in Fig. 3). (f,g) NC-AFM image simulation and (h) DFT geometry for model *E* with slightly higher energy and reduced match between experimental and simulated NC-AFM data. (i) Experimental XY image of CO type II^g (mirrored) and (k) experimental XZ scan from (b) (mirrored) to represent CO type II^g. (l,m) NC-AFM image simulation and (n) DFT geometry for model *F*. (o,p) NC-AFM image simulation and (q) DFT geometry for model *G*. Model *F* gives a similar agreement between theory and experiment as found for model *B*. In contrast, model *G* gives a reduced match between theory and experiment.

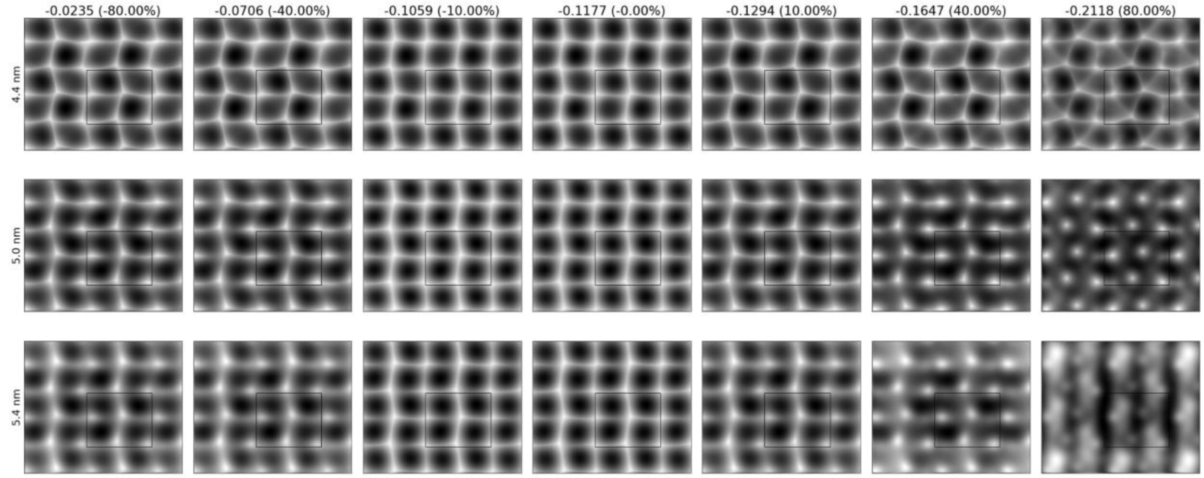

Figure S10:

Differences in the probe particle images with respect to a change in the charge of the oxygen atom in the CO molecule and at different heights. The probe particle images only showed translation transformations with a ±10% margin change in parameters determining the radial-stiffness, z-positions, tilt-angle of C and O in the tip CO molecule, the separation of metallic atoms in the tip, and the stiffness of the tip-apex. The features in the images, and their relative distances and angles, were found to be robust. In the case of charges, only varying the charge of the tip oxygen caused a significant change in the simulated image features, as shown in the figure. Within a ±10% change in charge, this change is limited to the sharpness of the features, and not their shapes, relative distances, or angles.

References

1. Heggemann, J.; Laflör, L.; Rahe, P. Double sample holder for efficient high-resolution studies of an insulator and a metal surface. *Review of Scientific Instruments* **2021,** *92* (5), 053705.
2. Tröger, L.; Schütte, J.; Ostendorf, F.; Kühnle, A.; Reichling, M. Concept for support and cleavage of brittle crystals. *Review of Scientific Instruments* **2009,** *80* (6), 063703.
3. Barth, C.; Henry, C. R. Kelvin probe force microscopy on surfaces of UHV cleaved ionic crystals. *Nanotechnology* **2006,** *17* (7), 155-161.
4. Kuhn, S.; Kittelmann, M.; Sugimoto, Y.; Abe, M.; Kühnle, A.; Rahe, P. Identifying the absolute orientation of a low-symmetry surface in real space. *Physical Review B* **2014,** *90* (19), 195405.
5. Rahe, P.; Schütte, J.; Schniederberend, W.; Reichling, M.; Abe, M.; Sugimoto, Y.; Kühnle, A. Flexible drift-compensation system for precise 3D force mapping in severe drift environments. *Review of Scientific Instruments* **2011,** *82* (6), 063704.
6. Giessibl, F. J. High-speed force sensor for force microscopy and profilometry utilizing a quartz tuning fork. *Appl. Phys. Lett.* **1998,** *73* (26), 3956-3958.
7. Albrecht, T. R.; Grütter, P.; Horne, D.; Rugar, D. Frequency-Modulation Detection Using High-Q Cantilevers for Enhanced Force Microscope Sensitivity. *Journal of Applied Physics* **1991,** *69* (2), 668-673.
8. Sugimoto, Y.; Nakajima, Y.; Sawada, D.; Morita, K.; Abe, M.; Morita, S. Simultaneous AFM and STM measurements on the Si(111)-(7x7) surface. *Physical Review B* **2010,** *81* (24), 245322.
9. Rahe, P.; Bechstein, R.; Kühnle, A. Vertical and lateral drift corrections of scanning probe microscopy images. *J. Vac. Sci. Technol. B* **2010,** *28* (3), C4E31-C4E38.
10. Nečas, D.; Klapetek, P. Gwyddion: an open-source software for SPM data analysis. *Central European Journal of Physics* **2011,** *10* (1), 181-188.
11. Lübbe, J.; Temmen, M.; Rahe, P.; Reichling, M. Noise in NC-AFM measurements with significant tip-sample interaction. *Beilstein Journal of Nanotechnology* **2016,** *7*, 1885-1904.
12. Kresse, G.; Furthmüller, J. Efficiency of ab-initio total energy calculations for metals and semiconductors using a plane-wave basis set. *Computational Materials Science* **1996,** *6* (1), 15-50.
13. Kresse, G.; Furthmüller, J. Efficient iterative schemes for ab initio total-energy calculations using a plane-wave basis set. *Physical Review B* **1996,** *54* (16), 11169-11186.
14. Bučko, T.; Lebègue, S.; Hafner, J. r.; Ángyán, G. J. Improved Density Dependent Correction for the Description of London Dispersion Forces. *Journal of Chemical Theory and Computation* **2013,** *9* (10), 4293-4299.
15. Tkatchenko, A.; Scheffler, M. Accurate Molecular Van Der Waals Interactions from Ground-State Electron Density and Free-Atom Reference Data. *Physical Review Letters* **2009,** *102* (7), 073005.
16. Bučko, T.; Lebègue, S.; Ángyán, G. J.; Hafner, J. r. Extending the applicability of the Tkatchenko-Scheffler dispersion correction via iterative Hirshfeld partitioning. *Journal of Chemical Physics* **2014,** *141* (3), 034114.
17. Grimme, S.; Antony, J.; Ehrlich, S.; Krieg, H. A consistent and accurate ab initio parametrization of density functional dispersion correction (DFT-D) for the 94 elements H-Pu. *Journal of Chemical Physics* **2010,** *132* (15), 154104.

18. Grimme, S.; Ehrlich, S.; Goerigk, L. Effect of the Damping Function in Dispersion Corrected Density Functional Theory. *Journal of Computational Chemistry* **2011,** *32* (7), 1456-1465.
19. Krukau, A. V.; Vydrov, O. A.; Izmaylov, A. F.; Scuseria, G. E. Influence of the exchange screening parameter on the performance of screened hybrid functionals. *Journal of Chemical Physics* **2006,** *125* (22), 224106.
20. Blöchl, P. E. Projector Augmented-Wave Method. *Physical Review B* **1994,** *50* (24), 17953-17979.
21. Hapala, P.; Kichin, G.; Wagner, C.; Tautz, F. S.; Temirov, R.; Jelínek, P. Mechanism of high-resolution STM/AFM imaging with functionalized tips. *Physical Review B* **2014,** *90* (8), 085421.
22. Hapala, P.; Temirov, R.; Tautz, F. S.; Jelínek, P. Origin of High-Resolution IETS-STM Images of Organic Molecules with Functionalized Tips. *Physical Review Letters* **2014,** *113* (22), 226101.
23. Di Giovannantonio, M.; Urgel, J. I.; Beser, U.; Yakutovich, A. V.; Wilhelm, J.; Pignedoli, C. A.; Ruffieux, P.; Narita, A.; Müllen, K.; Fasel, R. On-Surface Synthesis of Indenofluorene Polymers by Oxidative Five-Membered Ring Formation. *Journal of the American Chemical Society* **2018,** *140* (10), 3532-3536.
24. Liebig, A.; Hapala, P.; Weymouth, A. J.; Giessibl, F. J. Quantifying the evolution of atomic interaction of a complex surface with a functionalized atomic force microscopy tip. *Scientific Reports* **2020,** *10* (1), 14104.
25. Peng, J. B.; Cao, D. Y.; He, Z. L.; Guo, J.; Hapala, P.; Ma, R. Z.; Cheng, B. W.; Chen, J.; Xie, W. J.; Li, X.-Z.; Jelínek, P.; Xu, L.-M.; Gao, Y. Q.; Wang, E.-G.; Jiang, Y. The effect of hydration number on the interfacial transport of sodium ions. *Nature* **2018,** *557* (7707), 701-705.
26. Hämäläinen, S. K.; van der Heijden, N.; van der Lit, J.; den Hartog, S.; Liljeroth, P.; Swart, I. Intermolecular Contrast in Atomic Force Microscopy Images without Intermolecular Bonds. *Physical Review Letters* **2014,** *113* (18), 186102.
